# Obituary

**Published:** 1874-04

**Authors:** 


### OBITUARY.

Died recently, at Denver, Colorado, Mrs. A. M. Moore, wife of Dr. A. M. Moore, of Lafayette, Ind., aged 38 years.

Mrs. Moore had been an invalid since last May, and in November last went to Colorado. While there she rapidly regained health and strength, and expected soon to return to her home. But, a short time previous to her death, she heard of the death of a sister to whom she was most devotedly attached. She sank under the sad intelligence, and lingered but a day or two and died. Mrs. Moore was a most estimable lady and a dearly beloved wife and mother. Dr. Moore and his bereaved family will have the warmest sympathy of all acquaintances and friends.

